# IgG4-Related Disease Presenting as Isolated Scleritis

**DOI:** 10.1155/2017/4876587

**Published:** 2017-01-09

**Authors:** Eran Berkowitz, Ella Arnon, Alona Yaakobi, Yuval Cohen, Beatrice Tiosano

**Affiliations:** Department of Ophthalmology, Hillel Yaffe Medical Center Affiliated with The Bruce Rappaport School of Medicine, The Technion, Haifa, Israel

## Abstract

A rare case of IgG4-related disease (IgG4-RD) manifesting as nodular scleritis is presented in a 20-year-old female. Patient complained of left eye pain and redness for one week. Ocular examination together with ancillary testing led to the diagnosis of nodular scleritis. Since the patient did not show apparent improvement after one week of systemic steroidal treatment, she underwent a biopsy of the affected area revealing histopathological characteristics of IgG4-RD. Long-term treatment with corticosteroids and a steroid-sparing agent (methotrexate) led to significant improvement in signs and symptoms. This case highlights the significance of IgG4-RD in the differential diagnosis of scleritis and raises the question as to whether various organs affected by IgG4-RD may have different underlying pathophysiological mechanisms in which pathogenic T cells play a role.

## 1. Introduction

Immunoglobulin G4-related disease (IgG4-RD) is an immune-mediated condition that can affect almost any organ. The typical ophthalmic manifestation is lacrimal gland enlargement (dacryoadenitis). The diagnosis can be confirmed by blood tests and histology during the acute phase. We present a unique case of nodular scleritis due to isolated IgG4-RD, which responded well to immunosuppressive and immunomodulatory treatment.

## 2. Report of a Case

**Figure 1:**
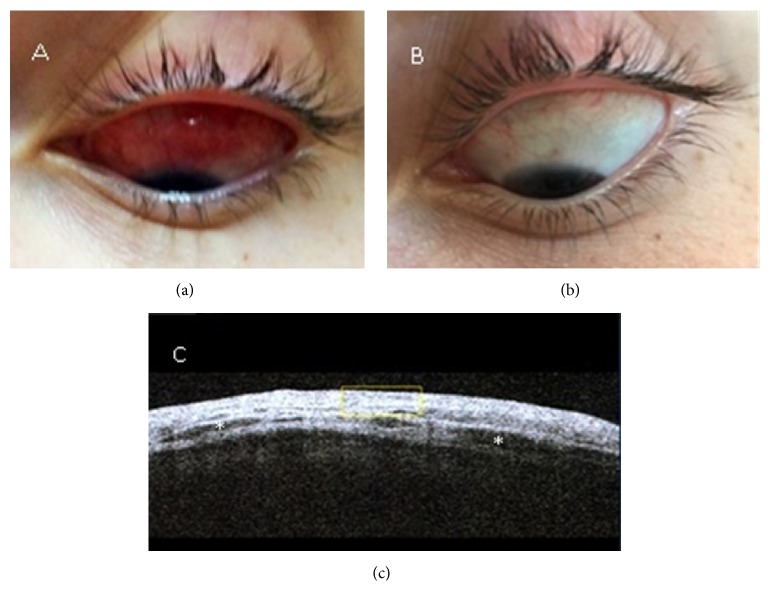
Color photograph of the superior bulbar area showing the area of inflammation before (a) and after (b) 5 weeks of treatment. Anterior segment ocular computerized tomography showing a thickened sclera and episclera with hyporeflective areas (asterisks) before treatment (c).

A 20-year-old female presented with complaints of discomfort and redness of her left eye of one week's duration. She had no associated visual complaints. Ocular examination revealed a localized inflamed superior bulbar conjunctival swelling in the form of scleritis. Ocular motility was preserved, and visual acuity was 6/6 in both eyes. Anterior segment ocular computerized tomography (OCT) demonstrated a thickened episclera and sclera with hyporeflective areas representing fluid in the region, confirming the diagnosis of scleritis (Figures 1(a) and 1(c)). Blood tests, including an autoantibody profile, were negative, and angiotensin-converting enzyme, thyroid function, and complement were normal. Kidney and liver functions were normal with no evidence of proteinuria. The serum IgG4 level was normal (97 IU/mL [normal < 121 IU/mL]), and the serum IgE level was elevated (371 IU/mL [normal < 300 IU/mL]). The chest X-ray and orbital magnetic resonance imaging study were noncontributory. She was diagnosed as having scleritis and received oral prednisone for 1 week, with no apparent response.

**Figure 2:**
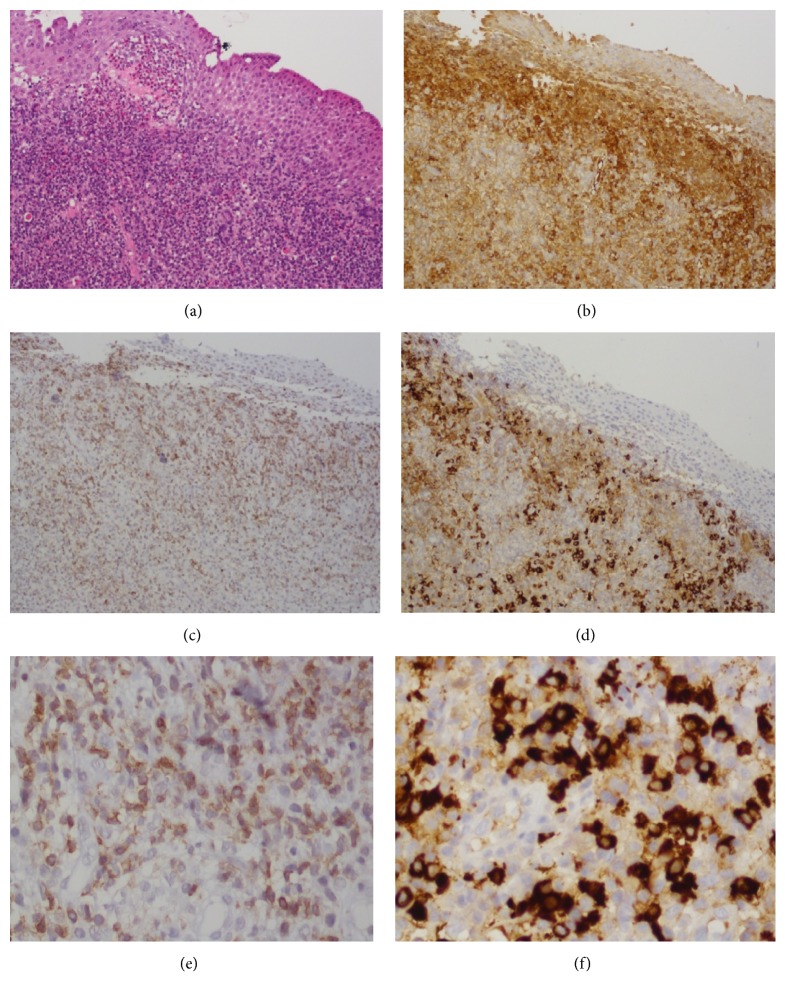
Histological Findings. (a) Hematoxylin-eosin stain at low-power field showing a marked lymphocytic plasmatic infiltrate. (b) Immunohistochemistry study for IgG. (c) Immunohistochemistry study for CD4. (d) Immunohistochemistry study of the same area demonstrating a diffuse pattern of IgG4-positive cells: a high proportion of the plasma cells are positive for IgG4 (~40%). (e) Immunohistochemistry study for CD4 cells at high-power field. (f) Immunohistochemistry study at high-power field showing >30 IgG4-positive cells per field.

The patient then underwent a biopsy of the affected area to confirm the diagnosis and to rule out conjunctival lymphoma. Histopathological analysis of the specimen revealed an intensive lymphoplasmacytic infiltration with a large number of IgG-positive plasma cells, >40% positive for IgG4. There were >30 IgG4 cells per high-power field. A large number of CD4+ positive cells (~50%) were observed in the specimen (Figures 2(a)–2(f)). A positron-emission tomography CT showed no other organ involvement. The patient was diagnosed as having IgG4-RD. Flow cytometric analysis of her peripheral blood revealed a normal cell expression profile (Table 1). Treatment with corticosteroids and a steroid-sparing agent (methotrexate) led to significant improvement in signs and symptoms, with the inflammation and swelling having almost completely subsided after 5 weeks of treatment (Figure 1(b)). The steroids were tapered down with no relapse of disease.

## 3. Discussion

Recurring anterior and posterior unilateral scleritis had been reported in a 63-year-old woman with a 13-year history of the disease [1]. And, to our knowledge, isolated anterior scleritis due to isolated IgG4-RD has not been previously reported. It is a systemic syndrome characterized by elevated serum levels of IgG4 and IgG4-positive lymphoplasmacytic infiltrations of organs, including orbital tissues. Approximately 30% of patients have normal serum IgG4 concentrations, despite classic histopathological and immunohistochemical findings [2]. Histopathological analysis of biopsy specimens remains the cornerstone of diagnosis, although there are subtle differences for different tissues [2, 3].

IgG4-RD can occur in any ocular adnexal tissue, for example, lacrimal glands, extraocular muscles, and cavernous sinus [4, 5]. The signs and symptoms of orbital IgG4-RD may be chronic lid swelling and proptosis, with only mild or no signs of inflammation or periocular pain [5]. There are two additional case reports on IgG4-RD involving the sclera: one described an IgG4-RD flare in a patient with a long history of the disease (diagnosis based on extraorbital biopsy, submandibular gland biopsy) [5] and the other was a case of IgG4-related pachymeningitis with concomitant scleritis, uveitis, and orbital soft-tissue involvement [6].

The biopsy in the case presented here met the criteria for probable IgG4-related inflammatory pseudotumor [3], which was used in the absence of specific criteria for IgG4-related scleritis. Biopsy is not, however, required for establishing the diagnosis of scleritis. The usual indication is to rule out an infiltrative process, for example, sarcoidosis or a lymphoproliferative disorder. Our patient's biopsy was performed to rule out lymphoma because of poor response to initial steroidal treatment.

CD4+ cytotoxic T cells appear to play an important role in disease pathophysiology. These cells have been shown to be dispersed throughout IgG4-RD lesions, as documented in our patient (Figures 2(c) and 2(e)). The clonally expanded population of CD4+ cytotoxic T lymphocytes in both the peripheral blood and the fibrotic lesions of IgG4-RD patients suggests that these cells are central to the disease. Mattoo et al. [7] reported that IL-1, TGF-beta, and interferon-gamma expressed by these cells are all potentially important mediators of the fibrosis which is a dominant part of the histopathology in IgG4-RD [7]. In the same work, the authors demonstrated that the number of these cells decreased concomitantly with a clinical response to rituximab therapy, suggesting a contributory role for these CD4+ cytotoxic T cells in the pathogenesis of IgG4-RD. Fibrosis and phlebitis were absent in histopathological specimens of the disease presenting as scleritis, as seen in the case report by Philippakis et al. [8] as well as in our case. Their absence may possibly hint at another pathophysiological pathway in various tissues where these CD4+ T cells play a different or a diminished role. In our patient, peripheral blood flow cytometry showed a normal T cell expression profile (Table 1), possibly explaining the absence of fibrosis in the specimens. It should be borne in mind, however, that the blood sample was taken a few months after starting treatment with methotrexate, an antimetabolite which affects both B cell and T cell populations by diminishing antigen-stimulated T cell proliferation and reducing peripheral blood T and B lymphocyte populations [9].

Long-term glucocorticoid treatment [2, 3, 5] is the first-line therapy for IgG4-RD. Therapeutic response is good, but significant relapse rates have been described following steroid discontinuation with a rise in the serum IgG4 levels, which necessitates a low maintenance dose of corticosteroids, possibly in combination with steroid-sparing agent, as was effective in the present case.

Based on these findings, we believe that the various organs affected by IgG4-RD may have different underlying pathophysiological mechanisms in which pathogenic T cell may play a different role.

## Figures and Tables

**Table 1:** Flow cytometric immunophenotyping of the patient's peripheral blood sample showing that 76% of the lymphocytes are T cells. Both the CD4/CD8 value and the T cell expression are within normal limits.

Antigen expression	Result (%)	Normal range
CD2	86	65–90
CD3	78	60–85
CD4	42	29–57
CD5	86	60–85
CD8	30	17–35
CD10	3	
CD56	17	5–15
